# Changes in dementia diagnoses in Sweden during the COVID-19 pandemic

**DOI:** 10.1186/s12877-022-03070-y

**Published:** 2022-04-26

**Authors:** Michael Axenhus, Sophia Schedin-Weiss, Lars Tjernberg, Anders Wimo, Maria Eriksdotter, Gustaf Bucht, Bengt Winblad

**Affiliations:** 1 Division of Neurogeriatrics, Department of Neurobiology, Care Sciences and Society, Center for Alzheimer Research, Karolinska Institutet, Solna, Sweden; 2 Theme Inflammation and Aging, Karolinska University Hospital, Huddinge, Sweden; 3 The primary care of Hudiksvall-Nordanstig, the Region of Gävleborg, Gävle, Sweden; 4 Division of Clinical Geriatrics, Department of Neurobiology, Care Sciences and Society, Karolinska Institutet, Solna, Sweden; 5 Department of Community Medicine and Rehabilitation, Umeå University, Umeå, Sweden

**Keywords:** Alzheimer’s disease, COVID-19, Dementia, Diagnosis, Sweden, Vascular dementia

## Abstract

**Introduction:**

The COVID-19 pandemic has caused large disruptions to healthcare systems. Refocus on COVID-19 related care might have contributed to indirect effects on other healthcare areas. Care focused on acute conditions have been negatively affected although research into the effects on chronic and care intensive patient groups such as patients with dementia diseases is lacking. In this study we evaluated dementia diagnosis trends in Sweden during 2015–2020 according to International Classification of Disease version 10 coding of common dementia diseases.

**Methods:**

Regional and national statistics in the form of International Classification of Disease version 10 coding, COVID-19 incidence, mortality data, and population census data were collected from the National Institute of Health and Welfare. Logistic regression analysis was performed to identify trends of dementia diagnosis during 2015–2020. Correlation test was performed between COVID-19 incidence, mortality rates, and dementia coding.

**Results:**

Dementia diagnosis incidence has been declining since 2015 and further decline was noted in many regions in Sweden during 2020. As COVID-19 incidence increased, fewer cases of dementia were diagnosed, a decrease that differentially impacted women and those who were advanced in age.

**Conclusions:**

Dementia diagnosis incidence in Sweden has been on a decline since 2015. The COVID-19 pandemic caused a further larger decline in dementia diagnosis incidence during 2020. COVID-19 incidence, but not mortality, was associated with decrease in dementia diagnosis incidence. There might be a large number of undiagnosed patients with dementia and healthcare reforms should be enacted to address this. Women and elderly are particularly vulnerable groups.

**Supplementary Information:**

The online version contains supplementary material available at 10.1186/s12877-022-03070-y.

## Introduction

The SARS-CoV-19 (COVID-19) pandemic has caused great disturbance to healthcare systems around the world [1, 2]. Many studies have reported lessened and worse quality of care for several acute conditions during the pandemic [3–5]. Changes in disease mortality trends have also been noted amongst several common diseases [6]. However, knowledge about the indirect impact of the pandemic on chronic conditions is not yet fully elucidated and the implications of healthcare disruptions amongst vulnerable groups is still an area of active research [7–9]. In order to formulate healthcare reforms and amend the damage caused by the pandemic, chronic and care-intensive patient groups needs to be studied in order to predict longitudinal effects.

An approximation of a specific disease burden can be obtained by studying the rate of healthcare visits per diagnosis in specialized out- and inpatient settings. The registration of specific International Statistical Classification of Diseases and Related Health Problems-Tenth revision (ICD-10) codes represents new diagnoses and follow-up visits and is indicative of the healthcare that a specific patient group receives. A decrease in ICD-10 coding therefore represents decreased care aimed towards a diagnosis. A significant disruption to care for a certain diagnosis group would be detectable in normal registry data. Vulnerable patient groups can be identified by categorizing diagnoses by age and sex. This knowledge is helpful in guiding future healthcare intervention and highlight the patient groups disproportionally affected by the pandemic.

Patients with dementia are some of the most care-intensive groups in health care systems. Diseases such as Alzheimer’s disease (AD), vascular dementia, and unspecified dementia take up large resources in the form of diagnostics and care. Physician’s visits, home care, long-term institutional care, and hospital stays for dementia patients are factors that account for a large portion of developed countries healthcare budgets [10, 11]. Despite this, dementia remains underdiagnosed [12, 13]. The increased implementation of acute care and change in clinical focus during the COVID-19 pandemic might have exacerbated the underdiagnosis of dementia and contributed to less care directed towards dementia populations. It is important to study the incidence of dementia diagnosis in specialized healthcare settings to quantify the pandemic impact on dementia patients.

In Sweden, healthcare systems rely on diagnostic coding, both in specialized out- and inpatient settings. Patient visits are coded per ICD-10 code according to the main reason for the visit. This coding includes both new diagnoses and follow up visits associated with that diagnosis. National statistics on the frequency of ICD-10 codes are available for both research and quality control purposes. Dementia diagnostics are interesting as they represent a significant healthcare burden both in both treatment and ongoing long-term patient care. Studies in other countries have shown that the COVID-19 pandemic has affected disease recognition and diagnosis, particular amongst age-associated illnesses in both primary care and hospital settings [14, 15]. The diagnosis of dementia has also been reported to have fallen drastically [14, 16, 17]. Furthermore, concerns have been raised regarding the disruption of care aimed towards noncommunicable and chronic diseases such as AD [18, 19]. The disruption to routine care for chronic health conditions will likely surpass the duration of the pandemic and have implications for years to come.

It exists no dementia incidence registry in Sweden but a quality-of-care registry, SveDem, register most new dementia diagnoses from both primary and specialist care. This data does not allow for incidence calculation but can be used to estimate if most dementia diagnosis is performed in either primary or specialist care.

There are indications of a decreasing incidence of dementia in Sweden [20, 21]. However, because of demographic trends with more elderly, the number of people with dementia is nevertheless increasing [22]. Thus, fewer dementia diagnoses as a result of the COVID-19 pandemic might lead to misrepresentation of disease burden caused by dementia, prompting research into this area.

By assessing the disease burden of dementia, measured as dementia-associated ICD-10 coding in out- and inpatient specialized care during 2020 compared to previous years, we aimed to evaluate how hard dementia diagnosis incidence were hit by the COVID-19 pandemic in Sweden. As various regions of Sweden report their ICD-10 coding separately, we furthermore aimed to study regional differences in dementia diagnosis incidence between the 21 regions of Sweden.

## Materials and methods

### Data acquisition

Sweden consists of 21 administrative regions; each one is responsible for healthcare within a designated geographical area. Oversight exists in the form of a central institution, the National Board of Health and Welfare, which register a multitude of data for quality assurance and research purposes. Among the parameters registered are ICD-10 codes. ICD-10 coding is used to quantify, amongst other things, the primary cause to why a patient required specialized care as well as new diagnoses. Data is reported by specialist care in both out- and inpatient settings.

We studied the reported incidence of dementia associated ICD-10 codes as described by the European shortlist of causes of death [23]. We included the ICD-10 codes for Alzheimer’s disease (G30), dementia concurrent with AD (F00), vascular dementia (F01), and unspecified dementia (F03) in the analysis. Using these codes, we collected all regional reported dementia cases reported per 100.000 inhabitants at the age of 65 or older. COVID-19 incidence, all-cause-, and dementia related mortality data per 100.000 were also retrieved from the database of the National Board of Health and Welfare. ICD-10 codes used for COVID-19 incidence included COVID-19-virus identified (U07.1) and COVID-19-virus not identified (U07.2). COVID-19 incidence was defined as number of cases per 100.000.

We collected all-cause mortality and dementia mortality. Mortality data was defined as the primary cause of death per 100.000. Dementia mortality included only deaths where the primary cause of death described on the death certificate was one of the previously described dementia codes. Data was obtained from the registers of the National Board of Health and Welfare and Statistics Sweden from 2015 to 2020 [24, 25].

We used SveDem data concerning newly registered dementia cases in primary or specialist settings from 2018 to 2020 in order to be able to compare between the two settings [26].

All data used were anonymized, publicly available and therefore not subjected to ethical review.

### Statistics

We used incidence of dementia diagnosis, measured as ICD-10 coding, from 2015 to 2019 to estimate the expected dementia diagnosis incidence for 2020. We deliberately chose to include only data starting at 2015 to emphasize recent trends in dementia diagnosis.

To estimate the predicted number of dementia diagnosis during 2020 we used logistic regression of the years 2015–2019 with the logarithm of dementia diagnosis as the dependent variable and the year as a continuous independent variable. The predicted number for 2020 was then calculated from the function equation and compared to actual outcome. To compare ratios of various dementia diagnosis in different settings, primary or specialist care, the same calculation was made with a logistic regression using the ratio of dementia diagnosis per setting as the dependent variable. Age cohorts were created, defined as dementia associated codes registered with people of either 65–74, 75–85, or 85+ years of age. The data was further subdivided in gender to assess sex specific differences. We analyzed the incidence for dementia diagnosis for 2020 by categorizing the data for each of the regions of Sweden in order to establish geographical differences. We used correlation analysis to determine the impact of COVID-19 incidence and mortality data on dementia diagnosis incidence. Correlation analysis was performed using Pearson correlation coefficient with a t-tailed t-test, *p*-value was considered significant at < 0.05. Data analysis was performed in SPSS version 28.0 (IBM, USA). All graphs were created using GraphPad version 9.1.1 (San Diego, CAL, USA).

## Results

There were significant regional differences in the incidence of dementia diagnosis when comparing expected to actual outcome. Most regions, 19 out of 21, reported a decrease in dementia diagnosis incidence during 2020 (Table 1).

**Table 1:** Regional differences in dementia diagnosis amongst the 21 regions in Sweden and the country as a whole. A majority of regions showed a significant decrease in dementia diagnosis

Region	2020	2020 expected incidence	Difference in expected vs actual incidence 2020 (%)	CI	P
Sweden	566,1	709,3	-20,20%	684–723	0,0043
Stockholm	1042,2	1392,9	−25,20%	1285–1476	0,0054
Uppsala	829,6	960,7	−13,60%	878–1093	0,0021
Södermanland	326,4	400	−18,40%	340–369	0,0054
Östergötland	517	510,4	1,30%	501–522	0,12
Jönköping	324,2	354,6	−8,60%	339–365	0,00098
Kronoberg	252,2	305,1	−17,30%	287–322	0,0032
Kalmar	341,6	432,6	−21,00%	407–463	0,0061
Gotland	400,4	527,2	−24,10%	501–549	0,0072
Blekinge	393,8	425,9	−25,40%	406–441	0,0012
Skåne	758,8	913,2	−16,90%	883–945	0,0038
Halland	307,6	316,5	−2,80%	301–329	0,063
Västra Götaland	280,8	381	−26,30%	356–402	0,0024
Värmland	257,5	277,5	−7,20%	260–291	0,0082
Örebro	462,8	575,5	−19,60%	553–597	0,0022
Västmanland	481,8	576	−16,40%	555–593	0,0019
Dalarna	448,8	615,7	−27,10%	594–635	0,0075
Gävleborg	349,9	534,7	−34,60%	502–567	0,000032
Västernorrland	559,2	680,3	−17,80%	661–699	0,0022
Jämtland	239,5	204,6	17,10%	192–224	0,0094
Västerbotten	931,1	1159,1	−19,70%	1052–1204	0,0068
Norrbotten	295,9	364,4	−18,80%	326–387	0,0046

Significant differences in expected dementia diagnosis incidence could be found in 19/21 regions. National change in the incidence for dementia diagnosis during 2020 was − 20%. Urban centers and northern rural areas showed the largest changes in dementia diagnosis incidence (Supplemental Fig. 1).

**Fig. 1:**
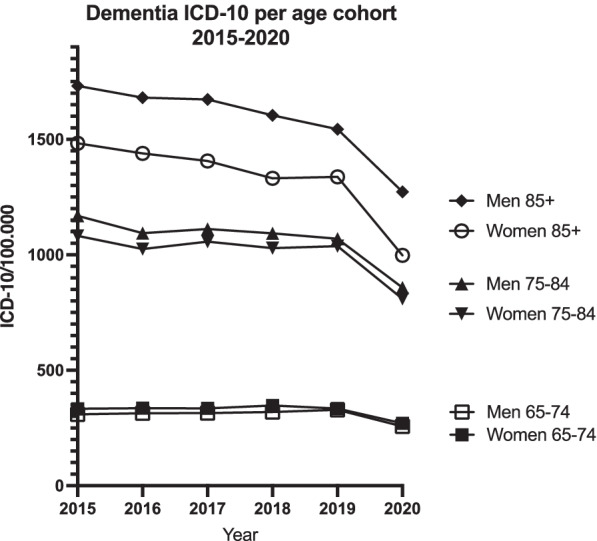
Dementia diagnoses trends per age and sex during 2015–2020. Whole year data indicated. Dementia diagnosis incidence has been slowly declining amongst elderly since 2015 with 2020 showing a further decrease in dementia diagnosis

Having found a significant drop in dementia diagnosis incidence during 2020, we wanted to investigate whether any patient populations were more affected than others. We therefore divided the data into age and sex groups in order to establish age or sex as a risk factor. Assessing the dementia diagnosis trends in Sweden during 2015–2019 showed no changes in dementia diagnoses for patients aged 65–74. For patient aged 74–85 or 85+, the incidence of dementia diagnosis has been slowly declining since 2015 (Fig. 1) (Supplemental Table 1).

There was no difference in dementia diagnosis incidence amongst men and women aged 65–74. In older groups, dementia diagnosis incidence has been declining during the last 5 years. 2020 marked a significant drop for all groups. Comparison between groups showed distinct differences (Table 2). Older age and female gender were associated with decreased dementia diagnosis incidence.

**Table 2:** Differences in dementia diagnosis incidence amongst age and sex groups. Elderly women were noted to experience a particular large decrease in dementia diagnosis

Group	2019	2020	Expected value 2020	Change in ICD-10 coding	CI	P
Men 65–74	328,4	256,6	320,8	−25%	301–340	0,0033
Women 65–74	334,2	270,3	338,7	−25%	321–342	0,0052
Both sexes 65–74	331,3	263,6	329,9	−25%	300–352	0,0029
Men 75–84	1069,3	857,6	1091,3	−27%	1002–1117	0,0017
Women 75–84	1037,5	809,5	1041,1	−29%	998–1093	0,0013
Both sexes 75–84	1052,2	832,1	1064,2	−28%	1003–1112	0,0019
Men 85+	1543,9	1272,5	1607,2	−26%	1504–1682	0,0015
Women 85+	1337,3	997,9	1358,6	−36%	1311–1398	0,00082
Both sexes 85+	1411,1	1096,6	1446,4	−32%	1384–1502	0,0012

**Fig. 2:**
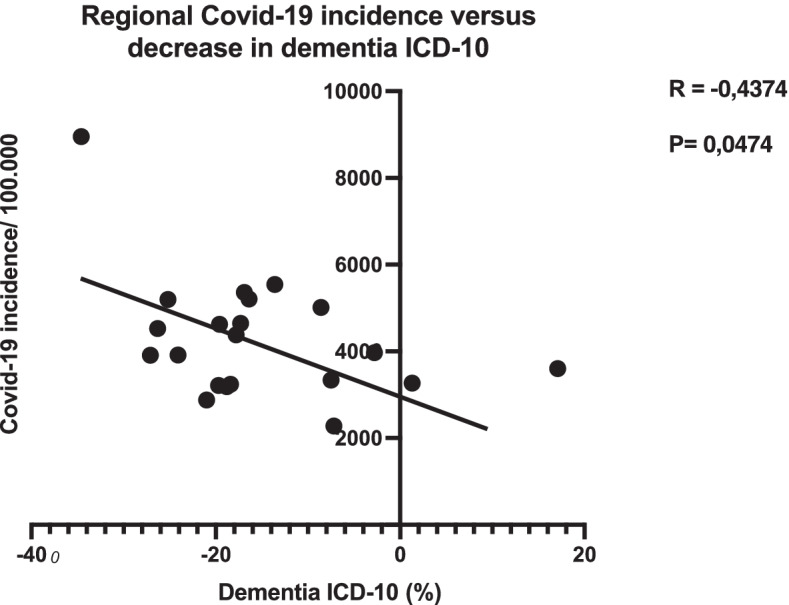
Regional COVID-19 incidence correlated to dementia diagnoses indicated that COVID-19 incidence caused a decline in dementia diagnoses throughout the country

2020 was a year of with many COVID-19 infections in Sweden and regions were hit unevenly. We correlated regional COVID-19 incidence as well as total mortality and dementia specific mortality against the incidence of dementia diagnosis to investigate whether COVID-19 infections or mortality drove dementia diagnosis decrease. Correlation analysis showed that as COVID-19 incidence increased, dementia diagnosis incidence decreased (Fig. 2). There was no correlation between all-cause mortality or dementia specific mortality (data not shown).

The majority of ICD-10 dementia codes were AD, with vascular dementia, and unspecified dementia making up the rest. We wanted to account for shifts in dementia diagnosis groups, such as AD ICD-10 coding transferring to unspecified dementia. We therefore compared the proportion of the various ICD-10 codes that were measured between 2019 and 2020. Whole nation data showed small ratio changes for AD and unspecified dementia with vascular dementia showing a ratio change of − 9.1% indicating a change in diagnostics aimed primarily away from vascular dementia (Table 3).

**Table 3:** Changes in percentages of dementia sub diagnoses making up dementia patient population. Large shift towards unspecified dementia and decrease in vascular dementia is noted, indicating a possible lack of quality amongst dementia diagnostics

Region	Alzheimer’s disease 2020	Vascular dementia 2020	Unspecified dementia 2020	Alzheimer’s disease 2019	Vascular dementia 2019	Unspecified dementia 2019	Change 2020 vs 2019 Alzheimer’s disease	Change 2020 vs 2019 Vascular dementia	Change 2020 vs 2019 Unspecified dementia
Sweden	65,70%	13,80%	20,50%	64,40%	15,20%	20,40%	2,00%	−9,10%	0,50%
Stockholm	72,40%	13,70%	13,90%	70,90%	15,80%	13,30%	2,10%	−13,10%	4,30%
Uppsala	59,40%	4,90%	35,70%	55,00%	4,20%	40,70%	7,90%	15,00%	−12,20%
Södermanland	46,80%	19,50%	33,60%	49,10%	21,10%	29,80%	−4,70%	−7,20%	12,80%
Östergötland	73,20%	11,10%	15,70%	71,10%	12,00%	16,90%	3,00%	−7,70%	−7,10%
Jönköping	58,90%	13,00%	28,00%	56,00%	14,80%	29,20%	5,20%	−11,90%	−3,90%
Kronoberg	38,90%	17,60%	43,50%	30,00%	22,90%	47,10%	29,70%	−23,00%	−7,70%
Kalmar	66,80%	12,30%	20,80%	58,10%	15,50%	26,50%	15,10%	−20,30%	−21,20%
Gotland	59,70%	29,00%	11,30%	64,00%	20,20%	15,70%	−6,80%	43,50%	−28,30%
Blekinge	60,70%	23,30%	16,00%	58,40%	22,60%	19,00%	3,90%	3,10%	−15,80%
Skåne	63,70%	15,90%	20,40%	66,20%	16,20%	17,60%	−3,80%	−1,70%	16,10%
Halland	54,90%	11,50%	33,60%	47,40%	19,40%	33,20%	15,80%	−40,70%	1,20%
Västra Götaland	56,70%	12,60%	30,70%	56,40%	12,70%	30,90%	0,50%	−0,40%	−0,70%
Värmland	57,70%	18,90%	23,40%	52,00%	19,40%	28,60%	11,00%	−2,70%	−18,20%
Örebro	61,60%	17,20%	21,20%	52,20%	22,50%	25,30%	18,00%	−23,60%	−16,10%
Västmanland	73,50%	10,30%	16,20%	68,20%	10,80%	21,00%	7,70%	−4,30%	−22,80%
Dalarna	72,10%	10,70%	17,20%	70,60%	14,50%	14,90%	2,10%	−26,30%	15,60%
Gävleborg	56,00%	21,40%	22,60%	56,10%	20,00%	23,90%	−0,20%	7,00%	−5,50%

**Fig. 3:**
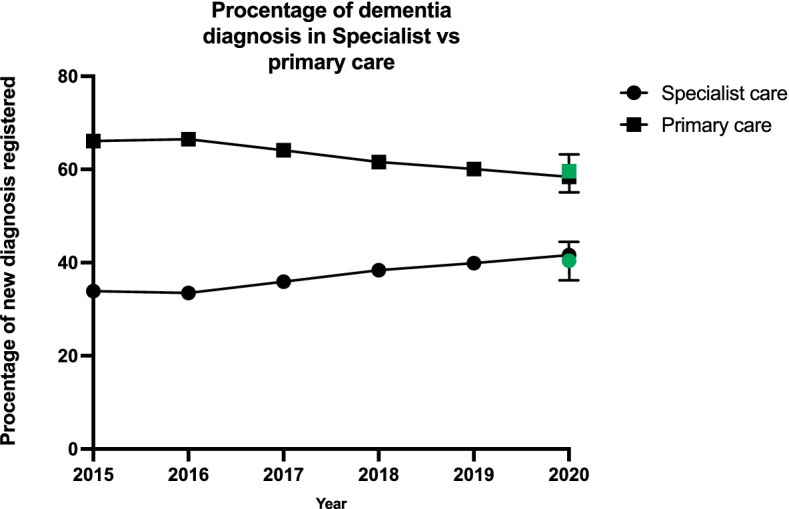
Percentage of dementia diagnoses in specialist and primary care. Dementia diagnoses percentage remained unchanged during 2020 compared to previous years. Green symbols represent predicted values for 2020

Pandemic conditions might cause dementia diagnoses to be allocated from specialized to primary care. No dementia incidence registry is available in Sweden although the Swedish registry for dementia, SveDem, can account for the location of dementia diagnosis, either in primary or specialist care. To account for changes in specialist versus primary care settings, the percentage of dementia diagnosis in primary care versus specialist care settings were compared. The ratio for 2020 did not differ from predicted values, indicating that a lack of diagnosis in specialist care settings could not be explained by an increase in primary care (Fig. 3).

## Discussion

The knowledge about the COVID-19 pandemic’s impact on chronic conditions such as dementia is lacking. The quantification of changes in dementia diagnosis incidence is essential in order to formulate future response and health reforms aimed toward the frail and elderly. In this study we compared ICD-10 dementia coding incidence in Sweden and its geographical regions in order to determine pandemic impact on dementia associated ICD-10 coding. We found a large reduction in dementia coding for the most common dementia codes throughout the country. This reduction was associated with regional COVID-19 incidence, with a higher COVID-19 incidence producing a larger decline in dementia diagnosis incidence. Elderly and women were groups that experienced disproportionally significant decreases in dementia diagnostics.

Our data showed that Swedish dementia diagnostics have been on a downwards trends the last few years, particular amongst elderly patient groups. This downward trend was exacerbated during the 2020 pandemic and resulted in a further decline in dementia diagnoses. Lack of knowledge regarding diagnosis criteria, refocus on other chronic conditions, and underfunded primary care system might be factors that contributed to this outcome. The need for dementia diagnosis is crucial and further highlighted by the COVID-19 pandemic. Patients with cognitive impairments are vulnerable to social isolation and decreases in mental stimulation. Isolation and decreased mental stimuli are both risk factors for dementia that have increased during the pandemic and directly lead to deterioration in cognitive function and increase the risk of dementia [27, 28].

Women and elderly in particular are at risk of dementia and represent vulnerable patient groups [29, 30]. This was also noted in our study with women and elderly patient groups having larger decreases in dementia diagnosis incidence when compared to other groups. It also appears that there are different levels of tolerance towards disruptions in diagnostics amongst the various diagnosis groups of dementia as we found vascular dementia cases to be more reduced compared to AD and unspecified dementia.

As ICD-10 coding does not differ between new diagnoses and follow up care, only expected incidence can be calculated. However, it is likely that both new diagnoses and follow up care are affected. This means that there is a significant underdiagnosis of dementia occurring in conjunction with the COVID-19 pandemic. This could mean that a large patient population is undiagnosed and left outside of the healthcare that they need, as patients with dementia are often dependent on services and special care. We suggest that healthcare systems should take actions to mitigate this shortcoming by allocating resources to primary care and geriatric clinics in order increase screening and diagnosis of new dementia cases. Decrease in registered dementia cases during 2020 might have been mitigated by the implementation of remotely accessible consultations, so called telemedicine, a strategy that has been applied in other countries [31–33].

The incidence of COVID-19 might skew statistics. As elderly are more susceptible to severe infection, a decrease in the total number of patients due to mortality might explain a decrease in dementia diagnosis incidence. We controlled for regional COVID-19 incidence and mortality, both all-cause and dementia. We found correlation between COVID-19 incidence and decrease in dementia diagnosis incidence. Regions with high COVID-19 incidence displayed larger decreases in dementia diagnosis. This is not surprising, given that a rerouting of resources and healthcare workers from a non-acute healthcare section to an acute one is to be expected. However, there was no correlation between mortality and dementia diagnosis incidence indicating that an increased mortality is not the cause of a decrease in dementia. This decrease is more likely driven by the overall healthcare burden on regional systems. If so, a continued dysfunction of dementia diagnosis can be expected for as long as the pandemic continues.

One further explanation for a decrease in dementia diagnosis incidence in specialist care could be an increase in primary care. This would occur as patients are being treated in primary care settings rather than being referred to specialist care. To account for this, we compared percentage of registered dementia diagnosis in primary versus specialist settings in a quality-of-care registry showing no significant change. This indicates that the lack of dementia care in specialist settings is not compensated by an increase in primary care. Together, these findings suggest that there are likely many undiagnosed and under-treated dementia patients in Sweden in the wake of the pandemic.

The results of this study are not surprising given that the COVID-19 pandemic and its subsequent restrictions has limited mobility of the elderly, likely leading to fewer doctor’s visits. Similar studies and reports in other countries have found results that are in line with our findings, showing large decreases in dementia diagnoses [14, 16, 17, 34]. Our study adds Sweden to the countries in which dementia diagnoses have been severely affected by the COVID-19 pandemic. If COVID-19 incidence is the driving factor behind decreased dementia diagnostics, then this pandemic might have worldwide ramifications for dementia patients and their caretakers.

Elderly patients are vulnerable to COVID-19 infections. Recommendations to stay at home and an unwillingness to expose oneself to hospital settings might be further contributing factors to decreased dementia diagnoses. Healthcare reprioritization in the form of relocation of healthcare resources from memory clinics and primary care centers is also likely to contribute to decrease in dementia diagnostics and treatments.

Due to the difficult and time-consuming process associated with confirming a dementia diagnosis, the drop in dementia diagnosis incidence might represent a significant loss in knowledge and experience amongst physicians treating dementia patients. Such a knowledge gap can prove disastrous going forward as the incidence of dementia might continue to decrease, leaving more and more patients without adequate care.

### Weaknesses

There are some weaknesses inherent with the data used in this study as it excludes private caregivers and does not differ between new diagnoses and follow-up visits. The data is assumed to be accumulative as more physicians are made aware of the registry as time goes on, making it difficult to draw conclusions about increases in dementia diagnosis incidence across regions. We were also unable to compare incidence of dementia in primary versus specialist care as such a dementia incidence registry does not exist in Sweden. Neither have we included data from the municipality sector, who has the main responsibility for long term care and home services.

## Conclusion

COVID-19 is correlated to decreases in dementia diagnoses as measured by ICD-10 coding. Elderly and women were particularly impacted with large decreases in dementia diagnosis incidence when compared to other groups. There is no evidence that a shift from specialist to primary care account for the lack of dementia diagnostics, indicating a large number of untreated and undiagnosed patients. Healthcare systems should carefully consider this lack of dementia diagnosis going forward.

## Supplementary Information

**Additional file 1: Supplementary Fig. 1.** Regional dementia coding in Sweden during 2020 compared to expected value. The largest decreases in the number of dementia diagnoses could be found in densely populated urban areas and northern rural areas.

**Additional file 2: Supplementary Table 1.** Dementia diagnoses per age and sex group during 2015–2020. A decline in dementia diagnoses can be detected in most groups even before the COVID-19 pandemic.

## Data Availability

All data is available from the authors on request. Primary ICD-10 coding data is freely available from the public records of the National Board of Health and Welfare. SveDem data is available from the SveDem website.
