# Supplementary material for: Changes in dementia diagnoses in Sweden during the COVID-19 pandemic
Source: BMC Geriatr. 2022 Apr 26;22:365. doi: 10.1186/s12877-022-03070-y (PMC9039601; doi:10.1186/s12877-022-03070-y)
Supplement: Supplementary file 1 — Additional file 1: Supplementary Fig. 1. Regional dementia coding in Sweden during 2020 compared to expected value. The largest decreases in the number of dementia diagnoses could be found in densely populated urban areas and northern rural areas. [file 12877_2022_3070_MOESM1_ESM.pdf]

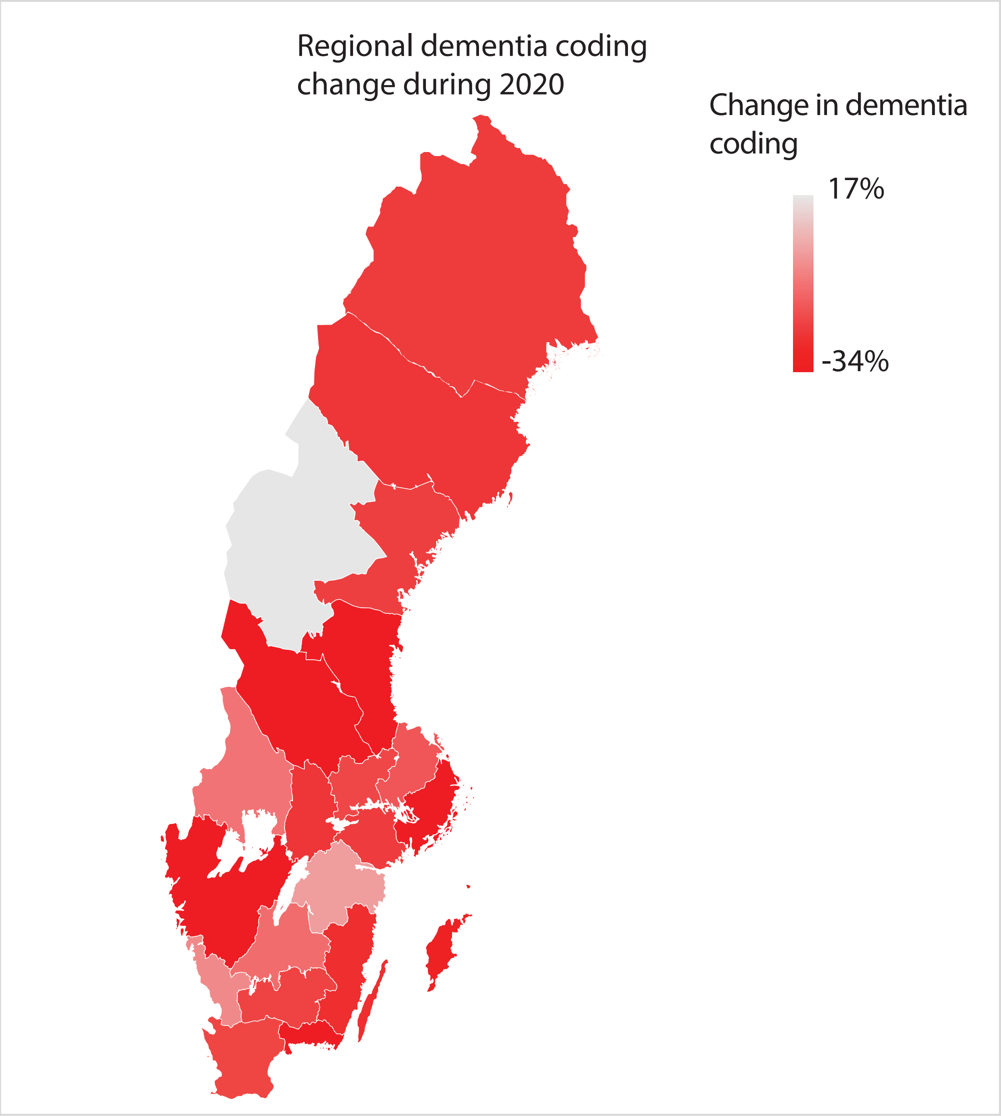

Supplementary figure 1: Regional dementia coding in Sweden during 2020 compared to expected value. The largest decreases in the number of dementia diagnoses could be found in densely populated urban areas and northern rural areas.
