# Supplementary material for: Changes in dementia diagnoses in Sweden during the COVID-19 pandemic
Source: BMC Geriatr. 2022 Apr 26;22:365. doi: 10.1186/s12877-022-03070-y (PMC9039601; doi:10.1186/s12877-022-03070-y)
Supplement: Supplementary file 2 — Additional file 2: Supplementary Table 1. Dementia diagnoses per age and sex group during 2015–2020. A decline in dementia diagnoses can be detected in most groups even before the COVID-19 pandemic. [file 12877_2022_3070_MOESM2_ESM.pdf]

	Dementia diagnoses/100.000						
	Men 65-74	Women 65-74	Men 74-84	Women 74-84	Men 85+	Women 85+	Men and women 65+
2015	309	334	1169	1082	1732	1483	6108
2016	314	336	1094	1025	1680	1440	5889
2017	314	335	1112	1057	1673	1407	5897
2018	320	347	1093	1029	1604	1332	5725
2019	328	334	1069	1038	1544	1337	5651
2020	257	270	858	810	1273	998	4464

Supplementary table 1: Dementia diagnoses per age and sex group during 2015-2020. A decline in dementia diagnoses can be detected in most groups even before the COVID-19 pandemic.
